# A New Compact Wideband Filter Based on a Coupled Stepped Impedance Resonator

**DOI:** 10.3390/mi15020221

**Published:** 2024-01-31

**Authors:** Abdel-Fattah A. Sheta, Ibrahim Elshafiey

**Affiliations:** Electrical Engineering Department, King Saud University, Riyadh 11421, Saudi Arabia; ishafiey@ksu.edu.sa

**Keywords:** compact structure, SIR filter, quarter-wavelength resonator, quarter-wavelength SIR, microstrip technology

## Abstract

A new compact wideband filter is introduced to address the requirements of recent communication and radar systems. The filter is based on a quarter-wavelength short-circuit coupled stepped impedance resonator (SIR). The analytical solution shows that the suggested SIR resonator provides a compact size and a wide stopband response, which are essential features in many wireless communication systems. The analytical results also reveal that increasing the impedance ratio of the SIR extends the stopband by increasing the first spurious response and reducing its total length. A compact two-coupled short-circuit SIR filter is designed at 1.23 GHz. The design approach is validated using an ideal transmission line modeling analysis and electromagnetic simulation using CST Microwave Studio 2021. The proposed structure is shown to be flexible, allowing the achievement of a relative bandwidth as low as 5% and as high as 50%. A four-resonator filter is designed by cascading two stages of the designed two-coupled short-circuit SIR filter, which are coupled through a quarter-wavelength line. The simulation results illustrate that the suggested structure can be used to design a filter with any number of resonators. The filter is implemented using a high-resolution LPKF laser machine on Rogers RT/duroid 6010.2LM material with a thickness of 0.635 mm. From the measurements, the bandwidth is found to be 390 MHz and centered at 1325 MHz (29.4% relative bandwidth) and the insertion loss is 1.3 dB. The simulation and experimental results verify the proposed approach and indicate the potential of this component in meeting the design requirements of next-generation microwave circuits related to flexibility and size-compactness.

## 1. Introduction

Recent advances in wireless communications for mobile, radar, and other various applications have raised the need for compact wide-band filters in the lower microwave range. Filters are extensively used in almost all wireless communication systems and are considered one of the key components in front-end modules. Typically, small-size, high-performance as well as low-cost filters are essential. Bandpass filters (BPFs) are used in transmitters and receivers. Their function in transmitters is to prevent any undesired signal produced by the nonlinear power amplifier from reaching transmitting antennas. On the other hand, filters protect receivers from noise or any undesired signal out of the band of interest in the space. Therefore, small-size, low-cost, as well as wide stopband characteristics with suitable frequency selectivity BPFs in transmitters and receivers are essential. Planar technology has many advantages that meet most of the filter requirements and provides a good tradeoff between performance, size, and cost [1,2]. Moreover, planar technology provides topological flexibility as well as adequate manufacturing control that leads to significant cost reduction. The most commonly used BPF configuration that is suitable for planar technology is based on half-wave and quarter-wave resonators. However, conventional uniform impedance resonators typically have limited stopband performance with a relatively large overall size. Various small-size planar filter techniques have been previously studied [3,4,5,6,7,8,9,10,11,12,13]. For compact requirements, dual-mode half-wavelength resonators in degenerate [3,4] or non-degenerate modes have been studied [5,6,7].

Filter structures based on dual-mode operation require half the number of resonators compared to conventional structures, which leads to small-size circuits. In degenerate modes, the two modes are orthogonal and have the same resonance frequencies. Filter response arises in a degenerate mode resonator by adding perturbation either by cutting or adding a small patch of square patch and ring resonator, respectively. This action allows the coupling between the two modes to take place and the resonator will be equivalent to two coupled single-mode resonator filters. These filters have a relatively narrow band response of <10%.

In a non-degenerate dual-mode filter, the response occurs by cutting symmetrical slots in the square patch, allowing the resonance frequency of the two first modes to be close to each other. These filters are suitable for a bandwidth that is approximately greater than 10% and less than 30%. Also, half-wavelength hairpin-line resonators have been structured in various topologies for compact configurations [8,9,10,11,12,13]. The parallel-coupled half-wavelength resonators are folded to form hairpin resonators as in [8,9]. A modified hairpin-line resonator is obtained by adding coupled lines at the open end for a greater size reduction, as suggested in [10], or by an arrangement in the form of cross-coupled lines as in [11]. In [12], a hairpin-line resonator is modified by a radial stub to improve the wide stopband characteristics. Recently, the half-wavelength wave resonator has been bending in a folded shape for compact applications [13]. The filter is designed in the S-band with a relative bandwidth of 10% with a significant size reduction. Another compact uniform half-wavelength open-loop resonator arranged in a coupled ring shape is suggested in [14]. These structures have limited stopband characteristics.

Half-wavelength SIRs have also been reported for small-size applications in [15,16]. A theoretical analysis showed that the physical length of the half-wavelength resonator can be decreased to one-half of its conventional length. Resonators are also bent to reduce the filter size. A half-wave SIR filter is also designed for wide stopband characteristics [17]. By adding appropriate input and output tapping, two transmission zeros can be located at the first spurious response to increase the wide stopband performance. Another half-wave SIR configuration is shaped in compact wide stopband performance [18] and used in high-performance triplexers [19].

Short-circuit quarter-wave resonators can also be used in bandpass filter designs [20,21]. Electric and magnetic coupling methods between quarter-wavelength SIRs are used to design compact bandpass filters [20] to provide quasi-elliptic characteristics. Quarter-wave SIRs based on coaxial resonators are firstly suggested for compact high-quality factor bandpass filters [22]. High characteristic impedance sections are used for interstage coupling.

Quarter-wave SIRs have been implemented in various topologies for many microwave applications [23,24,25,26,27]. Two short-circuit stepped impedance resonators are structured in a simple fashion for feeding and designed to eliminate the third harmonic [23]. The two-resonator filter is designed and implemented at 4.2 GHz with a relative bandwidth of 55%. In [24], stepped impedance short-circuit and open-circuit quarter-wavelength resonators are arranged to produce dual-band operation at frequencies of 2.45/5.25 GHz. The concept is used to design second and third-order BPFs at about 3.5 GHz. Also, dual-mode operation based on a short-circuit quarter-wavelength SIR filter is designed and implemented in [25] for operation at 2.4/5 GHz. Four resonators are used in this design and arranged in a compact structure. The quarter-wavelength SIR is also bent in a very compact shape and provides wide stopband characteristics [26]. In [27], a new coupling mechanism is introduced that allows for the design of filters with narrow and wideband performance. A short-circuit quarter-wavelength SIR is also suggested in [28] in a very compact spiral form at the cost of high insertion loss. In general, SIRs can be used as a compact part to design small-size components such as a triplexer [19], filtering power divider [29], and dual-band filters [30].

In this paper, a new short-circuit coupled quarter-wavelength SIR topology is introduced. The structure is arranged in a new compact form to meet the next-generation application requirements related to micro-machining and miniaturization. Besides its compact topology, the merit of the suggested approach lies in its flexibility to design filters with any order N and with relative bandwidths from 5% to 55%. Moreover, the suggested structure has very wide stopband bandwidth characteristics. A theoretical analysis of the short-circuit quarter-wave resonance features is provided in Section 2. A two-coupled resonator bandpass filter is presented in Section 3 to validate the suggested approach. In Section 4, the design and implementation of a four-resonator filter is presented to show the flexibility of improving filter response by cascading coupled resonators. Concluding remarks are given in the last section.

## 2. Resonance Characteristics of Quarter-Wave Resonator

**Figure 1:**
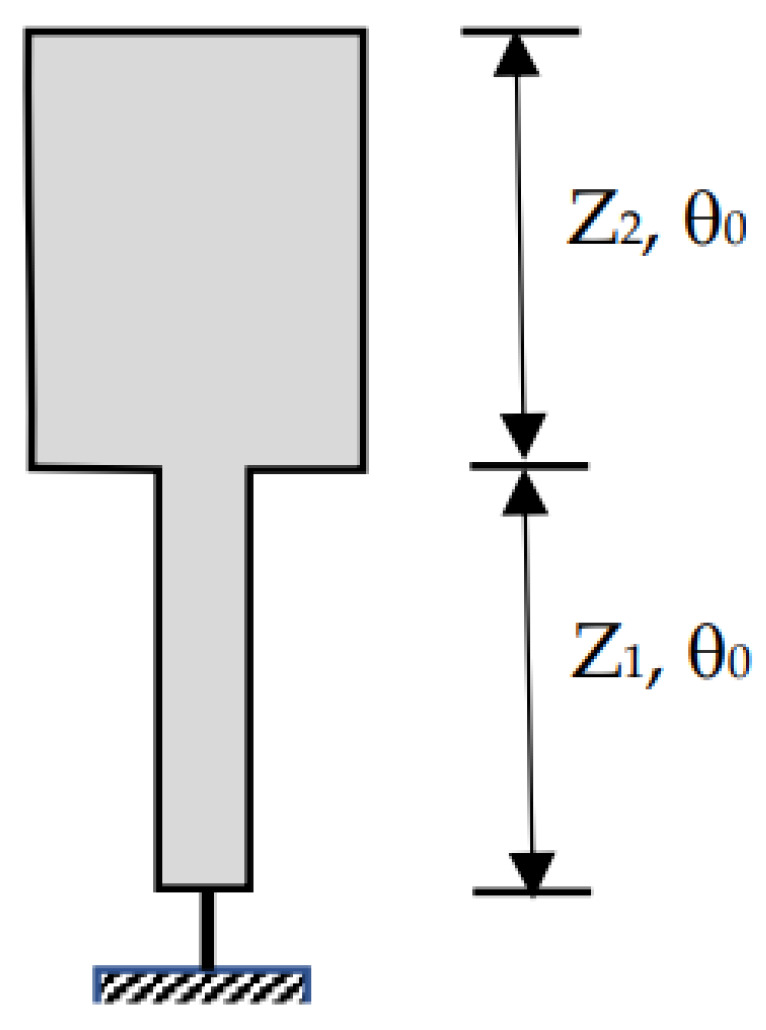
Short-circuit quarter-wavelength SIR.

The short-circuit stepped impedance resonator (SIR) considered in this paper is shown in Figure 1. The resonator consists of two transmission line sections of characteristic impedances Z_1_ and Z_2_. The two lines considered here are of the same electrical length, *θ*_0_, denoting the ratio Z_1_/Z_2_ = *K*.

At the fundamental resonance, the ratio *K* is related to the electrical length *θ*_0_ by the following equation:

(1) $$K=\cot^{2}\theta_{0}$$

It is clear that for *K* = 1, $\theta_{0}=\frac{\pi }{4},$ and the total electrical length = $2\theta_{0}=\frac{\pi }{2}$.

**Figure 2:**
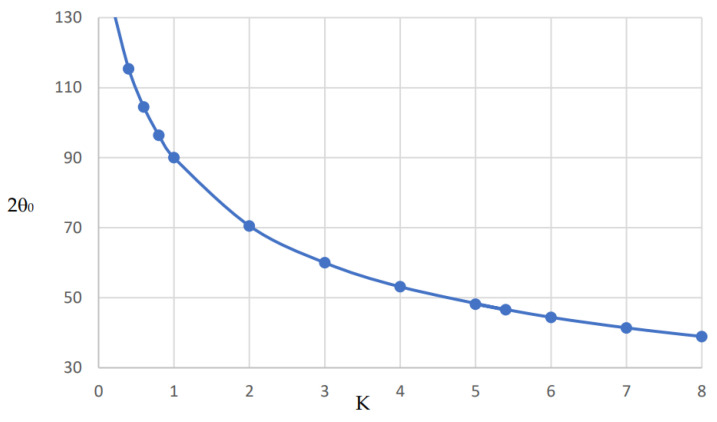
The total electrical length of the short-circuit resonator 2*θ*_0_ against *K*.

The plot of Equation (1) is shown in Figure 2. It is shown that as the ratio *K* increases, the resonator electrical length decreases. As an example, if the ratio *K* = 5, the total electrical length is about 48°. On the other hand, as *K* decreases, the resonator length increases. As numeric values, the total electric length is equal to 131.8° if *K* = 0.2. Therefore, for small-size circuits, a large value of *K* is recommended without violating the practical impedance limit of a given technology. At some conditions, large-sized circuits may be more appropriate according to the technology and performance requirements in the millimeter wave range, and in this case, *K* < 1 may be more suitable.

The spurious resonance frequency satisfies Equation (1). The first spurious resonance frequency denoting fs1 is related to the fundamental resonance frequency by the following equation:

(2) $$\frac{f_{s1}}{f_{0}}=\frac{\theta_{1}}{\theta_{0}}=\frac{\pi -\theta_{0}}{\theta_{0}}\;=\frac{\pi }{\theta_{0}}-1$$

In a similar way, the second spurious resonance frequency is also related to the fundamental frequency by the following equation:

(3) $$\frac{f_{s2}}{f_{0}}=\frac{\theta_{2}}{\theta_{0}}=\frac{\pi +\theta_{0}}{\theta_{0}}\;=\frac{\pi }{\theta_{0}}+1$$

**Figure 3:**
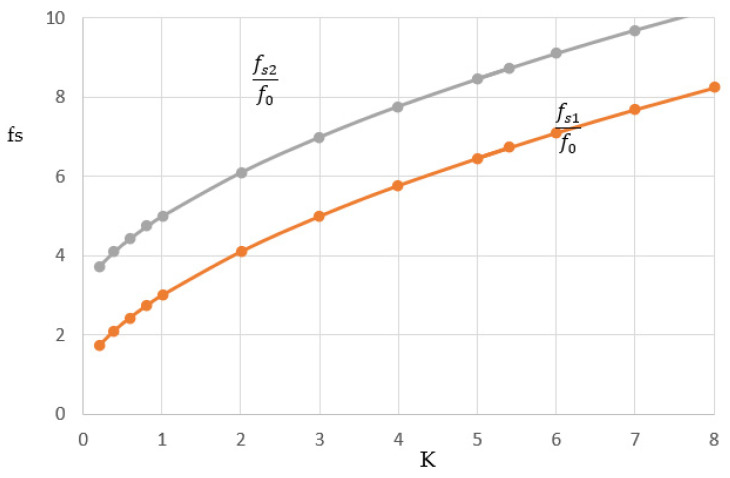
The plot of the first and second spurious resonance frequencies against K.

Based on Equations (1) and (2), the first and second spurious resonance frequencies, *f_s_*_1_ and *f_s_*_2_, are presented against *K*, as shown in Figure 3.

It is also observed that the spurious resonance frequencies *f_s_*_1_ and *f_s_*_2_ increase as the ratio *K* increases. For a uniform resonator, *K* = 1, the first spurious response will be at 3*f*_0_. Therefore, the stopband bandwidth lies between *f*_0_ and 3*f*_0_. Also, for K = 1, the resonator length will be 90°. For a *K* value greater than 1, the first spurious response increases and so the stopband bandwidth increases. However, increasing *K* is limited by the maximum and minimum characteristic impedances that can be implemented for a given technology and material. The microstrip line width decreases as the characteristic impedance increases and vice versa. Decreasing the line width increases the metallic loss and increasing the line width can increase the dielectric and radiation loss. For this reason, a tradeoff is needed for the optimum selection of *K*. Increasing the *K* value also decreases the resonator length, as depicted in Figure 2. For compact structure and practical reasons, *K* is recommended to be in the order of 5.

If *K* is selected to be 5, then we obtain *f_s_*_1_ = 6.47*f*_0_ and *f_s_*_2_ = 8.47*f*_0_. Therefore, using these parameters, we expect a very wide stopband in the upper frequency, as expected for a high impedance ratio. It is also evident that if *K* is less than 1, the first spurious response will be less than 2*f*_0_. The selection of *K* is also important to allocate the second band in case of dual-band operation.

The curves in Figure 3 are verified through the design of a resonator with Z_1_ = 50 Ω and Z_2_ = 10 Ω. The electrical length of each part is about 24.1°. Simulations have been performed using Keysight ADS 2021, where the line length is selected at a resonance frequency = 1.23 GHz. The first spurious resonance frequency is found at 7.91 GHz, and the second spurious resonance frequency is found to be 10.41 GHz, as expected from the curves.

## 3. Two-Resonator Filter Design

**Figure 4:**
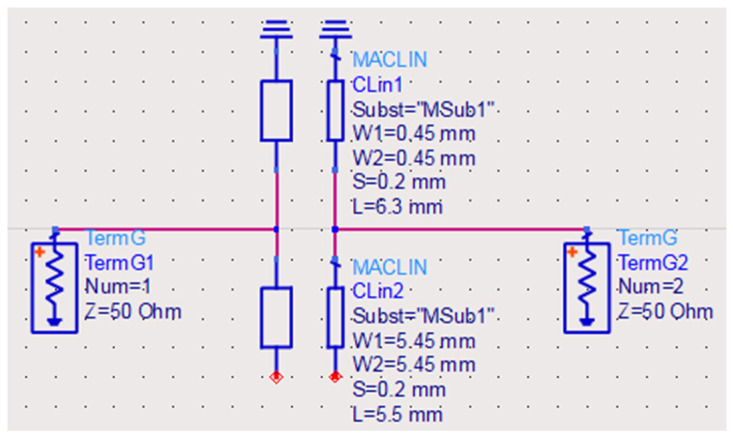
Schematic representation of a simplified coupled short-circuit quarter-wavelength SIR filter structure.

A simple filter consisting of two short-circuit coupled resonators based on the previously discussed structure is presented in this section. We maintain the same impedance ratio in the above example of *K* = 5, but in this case with practical TL. Rogers RT/duroid 6010.2LM material with ε_r_ = 10.2 and a thickness = 0.635 mm is used. The first short-circuit line section has a width = 0.45 mm and a length = 6.3 mm for 55 Ohms characteristic impedance (Z1) and 24.1° electrical length at 1.23 GHz. The second line has a width = 5.45 mm and length = 5.5 mm for 11 Ohms characteristic impedance (Z2) and 24.1° electrical length at 1.23 GHz. This filter is first simulated using ADS where feedings are directly connected at the contact between the two sections as shown on the circuit topology in Figure 4. The slot between the coupled lines is selected to be 0.2 mm.

**Figure 5:**
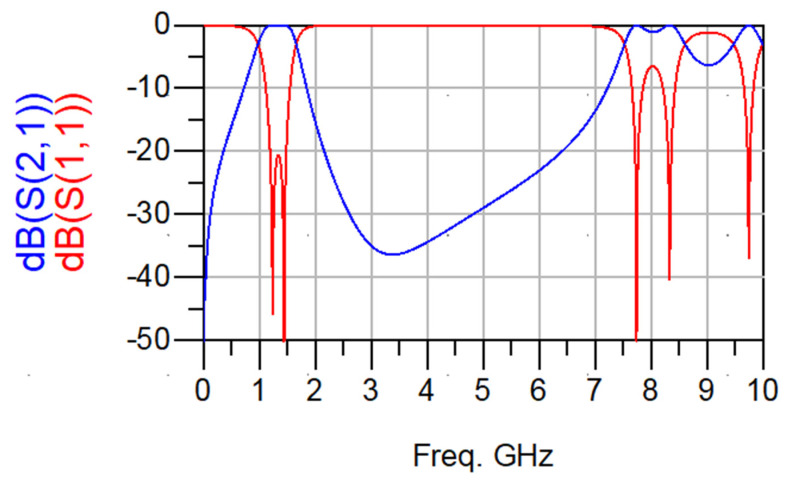
Keysight ADS simulation results of the two-resonator filter.

**Figure 6:**
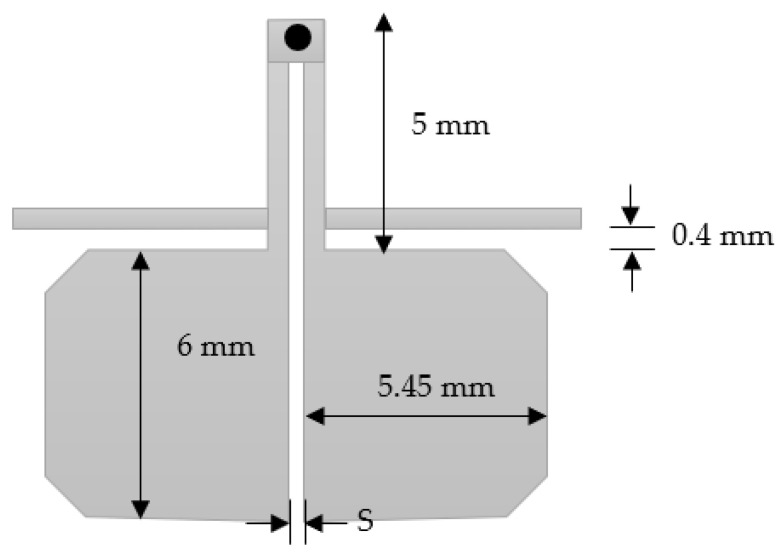
Two-coupled- resonator filter structure.

**Figure 7:**
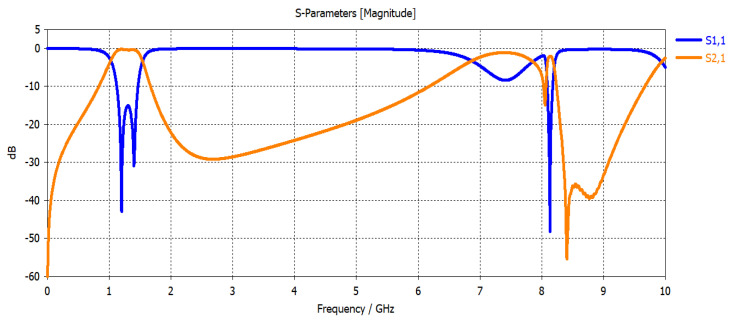
Simulation of the two-coupled short-circuit SIR filter using CST Microwave Studio.

From the simulation results shown in Figure 5, it is observed that the center frequency of the fundamental resonance is achieved approximately at the designed center frequency. The observed 3 dB bandwidth is about 600 MHz and centered at 1250 MHZ (48% relative bandwidth). This bandwidth decreases to 460 MHz if the slot width increases to 0.3 mm. As previously predicted, the first spurious response is also remarked at about 8 GHz, and the filter exhibits a wide stopband response. In addition, the investigation reveals that the center frequency is almost not affected by the slot width between the two coupled lines. Slot width affects the coupling and thus the bandwidth. The bandwidth extension can be achieved with the reduction of slot width. Also, the matching is highly affected by the slot’s widths. However, matching can be adjusted for any slot width through any matching procedure, such as quarter-wavelength feeding lines. This circuit is also simulated using CST Microwave Studio. In this case, minor adjustments are needed since the reference planes at which the feeding lines are connected and the effect of the step variation in width values will affect the results. The circuit topology is selected in such a way as to reduce the area of the filter, as shown in Figure 6. The slot width between the coupled lines is 0.3 mm, and the low-impedance lines have a width = 5.45 mm and length = 6 mm. The high impedance lines have a width = 0.45 mm and length = 5 mm. The CST simulation results of this configuration are shown in Figure 7. The filter is directly fed using 50 Ω characteristic impedance lines of a width = 0.45 mm as shown in Figure 7.

The filter exhibits a bandwidth of 390 MHz, centered at 1260 MHz (31% relative bandwidth). The reduction in bandwidth compared to the ADS simulation is due to a small adjustment in the resonator’s dimensions to accommodate the feeding lines and compensate for physical interconnections, reference planes, physical feedings, as well as the variation in step width as shown in the layout. The first spurious response is found at about 8 GHz.

**Figure 8:**
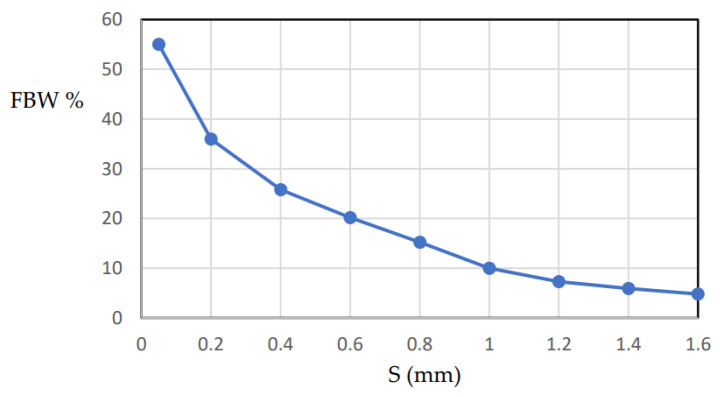
Fractional bandwidth against separation distance S.

The bandwidth is mainly determined by the following: (1) the coupling coefficients between the filter resonators; and (2) the external quality factor between the terminations and the adjacent resonators. The coupling coefficient and the external quality factor can be calculated based on the methods presented in [17,28]. The coupling coefficients between the coupled resonators depend on the separation distance S between the resonators. The external quality factor is determined by the position of the feeders along the first and last resonators. The feeder position can be optimized for bandwidth and matching. For design purposes, the fractional bandwidth (FBW) can be expressed as a function of the feeder position. For the direct feed two-resonator filter shown in Figure 6, the FBW is plotted against distance S, as shown in Figure 8. It is shown that the FBW increases as the separation distance decreases. The maximum FBW depends on the minimum separation that can be implemented according to the available technology. In our case, 55% FBW can be achieved for a minimum separation distance of 0.05 mm. In addition, 5% FBW can be achieved for S = 1.5 mm.

## 4. Four-Resonator Filter Design

The design of the four-resonator filter is studied in this section to show the flexibility of the proposed approach to design filters in any order. For simplicity, the four-resonator filter is designed by cascading two similar circuits of the presented filter in the previous section.

Coupled resonator filters can be designed based on the coupling coefficients and the external quality factor between each port and adjacent resonator as described in [13,17]. The design can be performed from the low-pass filter prototype. The external quality factor Qext and coupling coefficients can be related to the low-pass filter prototype parameters *g*_0_, *g*_1_, *g*_2_… *g_n_*, *g_N+1_* as follows.

For the first and last resonators, *Q_ext_* values are given by the following equation:

(4) $$\begin{array}{c} Q_{ext1}=\frac{g_{0}g_{1}}{FBW}\\ Q_{extN}=\frac{g_{N}g_{N+1}}{FBW} \end{array}$$

where *N* is the order of the filter. The coupling coefficients between resonators is given by the following equation:

$$K_{j,\;\;j+1}=\frac{g_{j}g_{j+1}}{FBW},\;J=1\;to\;N-1$$

For previously the discussed two-resonator filter, *N* = 2, it is designed by coupled symmetrical resonators. In this case, *g*_0_ = *g*_3_ and *g*_1_ = *g*_2_. These parameters are typical for a maximally flat frequency response filter where *g*_0_ = *g*_3_ = 1 and *g*_1_ = *g*_2_ = 1.4142

The design of a four-resonator filter, *N* = 4, can be carried out by cascading the previously designed two-resonator filter. Due to the symmetry, the following qualities must be satisfied: *g*_0_ = *g*_5_, *g*_1_ = *g*_4_, and *g*_2_ = *g*_3_. Also, a maximally flat response satisfies these qualities for *N* = 4 as follows: *g*_0_ = *g*_5_ = 1, *g*_1_ = *g*_4_ = 0.7654, and *g*_2_ = *g*_3_ = 1.8478. Therefore, cascading can be performed through the coupling between resonators two and three. The coupling is performed by a 50 Ω quarter-wavelength line as suggested and discussed in [28]. Very minor adjustments may be needed to compensate for the minor changes in *g*’s.

**Figure 9:**
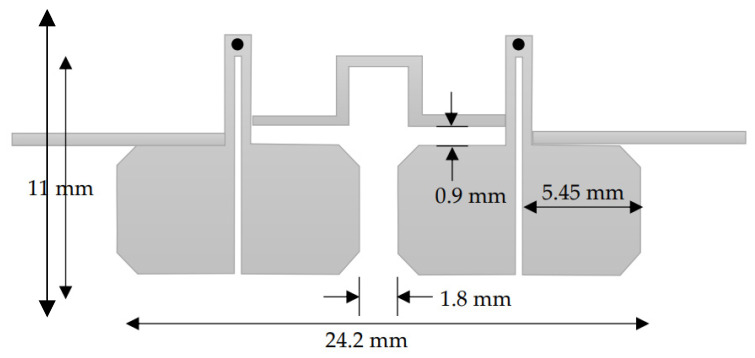
The layout of the four-resonator filter.

**Figure 10:**
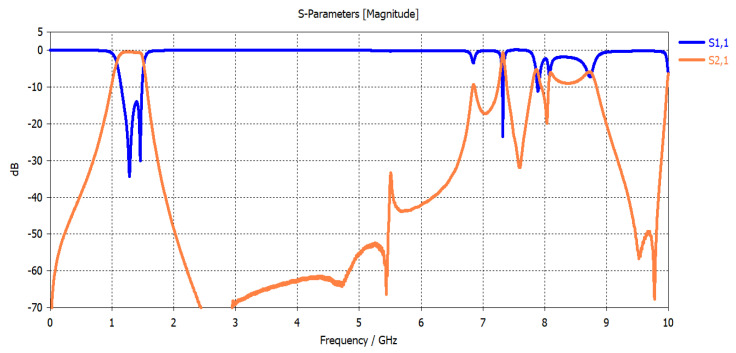
Simulation of the four-resonator filter using CST Microwave Studio.

The resultant circuit layout is shown in Figure 9. The simulation results using CST MW Studio are shown in Figure 10.

The simulated filter bandwidth is 360 MHz and centered at 1310 MHz (27.5% relative bandwidth). A minor reduction in the bandwidth compared to the two-resonator filter in Figure 6 is remarked, and this is due to a decrease in the low-pass filter prototype parameters in the case of the fourth-order filter. For this reason, a minor adjustment in the coupling is added. The simulated insertion loss is found to be 0.7 dB. Also, a minor shift of the center frequency is observed, which may be attributed to the modification of the input/output feedings as shown in Figure 6.

**Figure 11:**
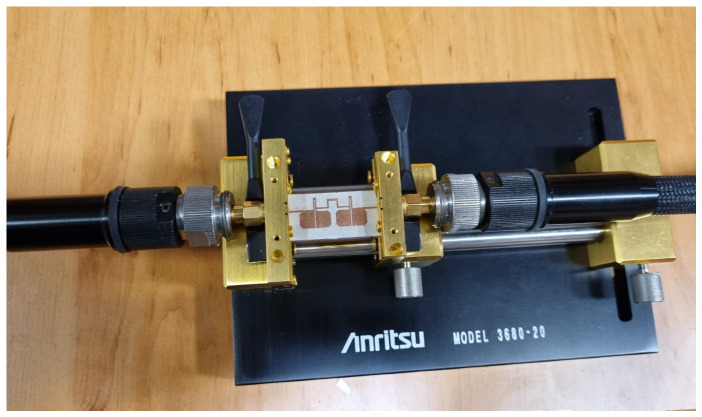
Photograph of the fabricated four-resonator filter hold on Anritsu test fixture 3680-20.

**Figure 12:**
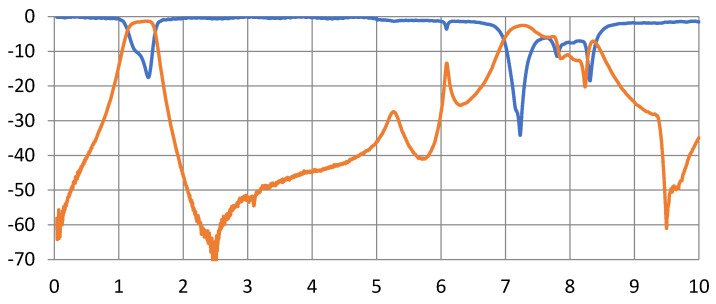
Measurements of the fabricated four-resonator filter showing S11 (blue) and S21 (brown).

This circuit is implemented on the same substrate using the LPKF Laser and Electronics, Germany, machine with a minimum line width and separation between the line’s capability of 50 µm. Figure 10 shows a photograph of the implemented filter. The size of the filter is 11 × 24.2 mm^2^. This significant size reduction of the four-resonator filter at 1.31 GHz is achieved by the selection of high dielectric constant material and the short-circuit stepped impedance resonator. The filter is experimentally characterized using the Vector Network Analyzer 37369C, and held in the test fixture 3680-20, both from Anritsu, Japan, as shown in Figure 11. The measurement results are shown in Figure 12. The measured 3-dB bandwidth is 390 MHz, centered at 1325 MHz (29.4% relative bandwidth). The insertion loss is measured to be 1.3 dB. The measured insertion loss is higher than the simulated one by about 0.6 dB, which may be attributed to some leakage of the power at the connections to the test fixture. A minor decrease in the rejection in the stopband is observed from 2 to 6 GHz. This may be attributed to the test fixture structure since its walls are very close to the resonators. Still, the rejection is more than 40 dB from 2 to 5 GHz, and greater than 27 dB from 5 to 6 GHz. Overall, these results are very close to the predicted results shown in Figure 10.

A comparison of the proposed filter structure and other designs found in the literature is shown in Table 1. References [13,14,15,16,17,18,19] in Table 1 present resonators that are based on a half wavelength either in an open loop or SIR shape. All designs have relatively narrow-band, large-size, and low-stopband bandwidth characteristics compared to the filters based on the quarter-wavelength SIR in [20,26,27,28]. Small-size and low-IL circuits are usually contradicted. Although some filters have smaller sizes compared to the suggested structure of this work, as in [26,28], they have higher IL. So, the existing work provides a very small size with reasonable IL.

## 5. Conclusions

A new quarter-wavelength short-circuit coupled SIR filter topology has been introduced, designed, simulated, and experimentally validated. The filter is based on a compact short-circuit quarter-wavelength SIR. A filter based on two coupled resonators of this type is designed and simulated using ideal TL sections to validate the concept. Keysight ADS simulator is used in this step. The filter is directly fed at the interface between the high- and low-impedance sections. This filter is also electromagnetically simulated, and some minor adjustments have been performed to compensate for physical interconnections, reference planes, physical feedings, as well as the variation in step width. The flexibility of the new design approach allows us to design filters with any order N with relative bandwidths from 5% to 55%, and maintain a very wide stopband bandwidth. The four-resonator filter is designed by cascading two identical elements of the two-coupled resonator filter. The coupling between the two circuits is performed using a quarter-wavelength line. This filter is implemented on Rogers RT/duroid 6010.2LM material with ε_r_ = 10.2 and thickness = 0.635 mm. The experimental characterization is achieved using the Anritsu Vector Network Analyzer 37369C and held in a test fixture. The measured bandwidth is 390 MHz and centered at 1325 MHz (29.4% relative bandwidth). The insertion loss is measured to be 1.3 dB. The wide stopband is remarkable since the first spurious response is noticed at around 8 GHz. A relative bandwidth from 5% to 50% can be achieved by the proposed structure through the control of the slot width between the coupled lines. The design process should consider enhancing the filter performance by increasing the number of elements while satisfying space limitations mandated for microwave circuits in next-generation systems.

## Figures and Tables

**Table 1:** Comparison between the proposed filter and related filter designs in the literature.

Reference Number	h (mm)	ε_r_	*f*_0_ (GHz)	FBW %	IL (dB)	Size (λg × λg)	# of Resonators
[13]	1.27	10.8	2.2	12.5	0.9	0.41 × 0.08	5
[14]	0.787	2.2	3.5	2.8	2.67	0.4 × 0.4	4
[17]	0.508	2.2	1.5	6	2.2	0.136 × 0.5	3
[18]	1	4.4	1	12	3.5	0.215 × 0.22	4
[19]	0.508	3.38	1.47	4.96	3.2	0.25 × 0.234	4
[20]	0.508	2.2	3.5	8.5	1.2	0.1 × 0.19	3
[26]	0.508	3.38	2.01	12.6	2.5	0.2 × 0.13	4
[27]	0.813	3.38	2	5.1	2.32	0.085 × 0.14	2
[28]	0.508	3.38	1	24	2.61	0.12 × 0.06	6
This Work	0.635	10.6	1.3	28	1.3	0.12 × 0.26	4

## Data Availability

Data is contained within the article.
